# P-737. Highly Resistant *Myroides* Species Infections: Insights from a Comprehensive Case Series and Antibiogram Analysis

**DOI:** 10.1093/ofid/ofae631.933

**Published:** 2025-01-29

**Authors:** Avi Toiv, Neal Doshi, Angela Ishak, Odaliz Abreu Lanfranco

**Affiliations:** Henry Ford Hospital, Detroit, Michigan; Michigan State University College of Human Medicine, Detroit, Michigan; Henry Ford Hospital, Detroit, Michigan; Henry Ford Health System, Wayne State University School of Medicine, Detroit, MI

## Abstract

**Background:**

*Myroides* species (spp.) are rare opportunistic gram-negative bacteria that are highly resistant to commonly used empiric antibiotics. Able to survive in natural and hospital environments, these microbes pose a growing risk of resistant nosocomial infections. Here we present a case series and antibiogram of *Myroides* infections at an academic medical center, highlighting this potential pathogen’s antibiotic sensitivity to aid clinicians in choosing effective antimicrobial therapies.

Antibiogram

**Figure:**
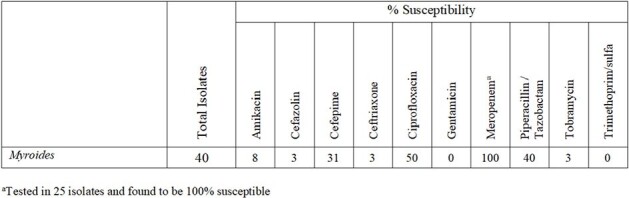

**Methods:**

Retrospective chart review of all patients diagnosed with *Myroides* spp. infection at Henry Ford Health between January 1, 2019 and December 31, 2023. Patient characteristics, treatments, and outcomes were analyzed. An antibiogram of *Myroides* spp. susceptibility to 10 antibiotics was generated.

**Results:**

A total of 43 patients (median age 62 yr; range 30-94) presented with 46 *Myroides* spp. infections. Positive cultures were evenly distributed between hospitalizations (50%) and outpatient (50%) settings. Of the 46 infections, 35 (76%) were in a lower extremity wound. Of the 43 patients, 17 (40%) had sepsis at presentation, including 8 (13%) with *Myroides* bacteremia. Most *Myroides* spp. cultures (83%) grew additional organisms, including *E. faecalis* (34%), *S. aureus* (21%), *Proteus mirabilis* (21%), and *Pseudomonas spp*. (16%). Empiric antibiotics were prescribed in 35/46 cases (76%), mostly vancomycin (17/35) and cefepime (9/35); however, *Myroides* spp. were sensitive to the empiric agents in only 4 cases (11%). Antibiogram analysis showed 100% susceptibility to meropenem and 0%-50% susceptibility to 9 other agents (Table). A total of 5 (12%) patients died, and 6 patients (14%) required readmission within 1 month of treatment.

**Conclusion:**

*Myroides* spp. exhibited significant resistance to most empiric antibiotics. Our antibiogram analysis revealed meropenem as the sole effective empiric antibiotic for this opportunistic pathogen. We recommend the prompt empirical use of meropenem when *Myroides* spp. are identified on antimicrobial culture.

**Disclosures:**

**All Authors**: No reported disclosures

